# Pilot Study Using a Chitosan-Hydroxyapatite Implant for Guided Alveolar Bone Growth in Patients with Chronic Periodontitis

**DOI:** 10.3390/jfb8030029

**Published:** 2017-07-19

**Authors:** Fabiola Vaca-Cornejo, Héctor Macías Reyes, Sergio H. Dueñas Jiménez, Ricardo A. Llamas Velázquez, Judith M. Dueñas Jiménez

**Affiliations:** 1 Department of Physiology, University Center of Health Sciences ,University of Guadalajara, Guadalajara, Jalisco 44100, Mexico; sidlc@hotmail.com (F.V.-C.); sduenas@cucs.udg.mx (S.H.D.J.); 2 Department of Clinical Surgery, Fray Antonio Alcalde Hospital, University of Guadalajara, Guadalajara, Jalisco 44100, Mexico; hectormaciasr@gmail.com; 3 Sophia Laboratories, Guadalajara 45010, Jalisco, México; drricardollamas@gmail.com

**Keywords:** chitosan, hydroxyapatite, periodontitis, guided bone regeneration

## Abstract

Periodontitis is an infectious and inflammatory disease associated with significant loss of alveolar crest and soft tissue attached to the teeth. Chitosan and hydroxyapatite are biomaterials used for bone tissue repair because of their biodegradability and biocompatibility in nature. The present study evaluated the effects of chitosan (CH) in combination with hydroxyapatite (HAP) to promote alveolar bone growth. A chitosan implant mixed with hydroxyapatite was implanted into the affected area of 9 patients suffering chronic periodontitis. Patients were evaluated through X-ray images and a millimetric slide over a one year period. The application of CH/HAP produced an average alveolar bone growth of 5.77 mm (±1.87 mm). At the onset of the study, the dental pocket exhibited a depth level (DPDL) of 8.66 mm and decreased to 3.55 mm one year after the implant. Tooth mobility grade was 2.44 mm at the onset and 0.8 mm at the end of the study with a significant difference of *p* < 0.001. Moreover, the bone density in the affected areas was similar to the density of the bone adjacent to it. This result was confirmed with the software implant viewer from Anne Solutions Company. In conclusion, the CH/HAP implant promoted alveolar bone growth in periodontitis patients.

## 1. Introduction

Chronic periodontitis is caused by aerobic and anaerobic microorganisms. Clinical manifestations depend on the host´s response to the bacterial invasion. Chronic periodontitis also produces bone loss as well as damage to the soft tissue attached to it, which is accompanied by an inflammatory tissue reaction and extracellular matrix breakdown. At the present time, the most common periodontitis treatment is Emdogain, a protein derived from developing pork enamel. This protein promotes alveolar bone growth and the periodontal mucous membrane restitution [1]. The combined treatment of Emdogain with the bovine lyophilized bone protein has a better outcome compared to individual treatments [2]. However, this combined treatment is expensive for most people living in low-income countries. An alternative inexpensive treatment is guided bone regeneration. It consists in the insertion of a membrane separation between the soft tissue and the bone defect in order to allow bone regeneration and thus, avoid mucous invagination [3].

A 3D scaffold embedded in a synthetic extracellular matrix (ECM) environment could be a good biomaterial to produce tissue regeneration. Furthermore, it should be biodegradable, easy to apply and effective for bone repair [4]. Chitosan is a partially N-deacetylated biomaterial, a derivative of chitin (2-acetamido-2-deoxy-β-d-glucose through a β (1–4) linkage). Macrophages, convert chitosan into chitooligomers. In vivo, these oligomers stimulate fibroblasts to produce collagen and ECM [5]. The combination of collagen and hydroxyapatite Ca_10_(PO4)_6_(OH)_2_ works as a guide for converting chitosan into an osteoinductor [6]. In rats, powder chitosan has been used as a material to fill a tibia bone defect, where bone repair was faster in the chitosan group compared with the control group after four weeks of treatment. This biomaterial was also used to promote human bone repair [7]. Hydroxyapatite has also been used as a substitute in bone grafting [8] and it is designed to work as a temporary template for migration, proliferation, and differentiation of osteoblasts. Calcium deficient hydroxyapatite (CDHA) and silicon substituted hydroxyapatite (SiHA) macroporous scaffolds favor the adequate interaction with osteoblast-like cells and pre-osteoblast-like cells producing a higher bone cell proliferation and differentiation [9]. 

The effects of the combination of HAP and chitosan to promote alveolar bone regeneration in local chronic periodontitis patients are unknown. Therefore, the present study evaluates chitosan in combination with hydroxyapatite in guided bone regeneration of periodontitis patients. 

## 2. Results

**Figure 1:**
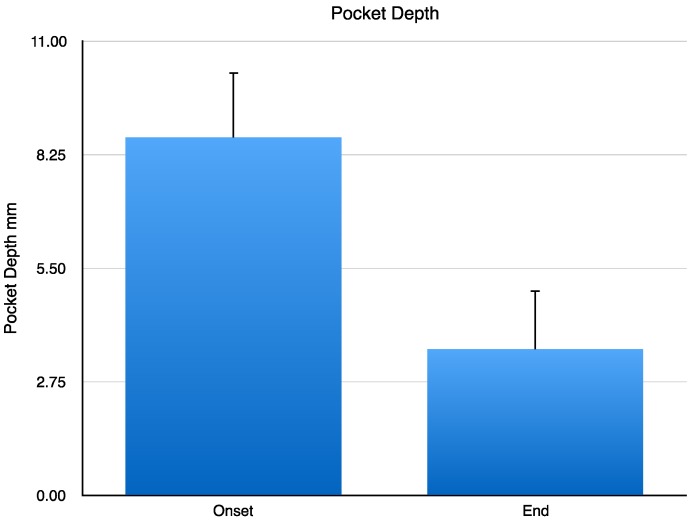
Pocket depth (mm) in patients with chronic periodontitis. Data are expressed as mean ± standard deviation (X ± SD). Left bar shows the pocket depth (mm) in periodontitis patients measured at the onset of the study, and the right bar exhibits the pocket at the end of the study. Data were analyzed by Student’s *t*-test. This change has a statistically significant difference of *p* < 0.001 (Figure 1).

At the onset of the clinical study, nine patients with chronic periodontitis exhibited a dental pocket depth level (DPDL) of 8.66 ± 0.52 mm. A year after the implant with CH/HAP, DPDL changed to 3.55 ± 0.47 mm. Data were analyzed by Student’s *t*-test. This change has a statistically significant difference of *p* < 0.001 (Figure 1).

**Figure 2:**
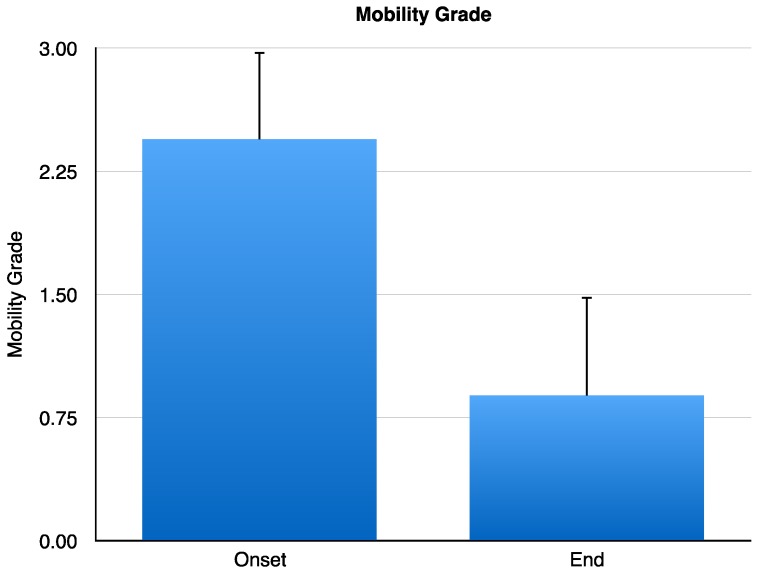
Tooth mobility grade (mm) in periodontitis patients. Data is shown as mean ± SD and the results were compared at the onset and at the end of the study. Mobility grade in pp at the onset of study was of 2.44 mm ± 0.1757 and at the end of the end of the evaluation it decreased to 0.8889 ± 0.2003 mm (gray bar) with a statistically significant difference of *p* < 0.001 evaluated by Student’s *t*-test. SD: Standard Deviation.

At the onset of the study, tooth mobility grade was of 2.44 ± 0.17 mm, and one year after CH/HAP treatment the dental mobility grade was reduced to 0.88 ± 0.20 mm. This reduction has a statistically significant difference of *p* < 0.001 (Student’s *t*-test) (Figure 2). 

**Figure 3:**
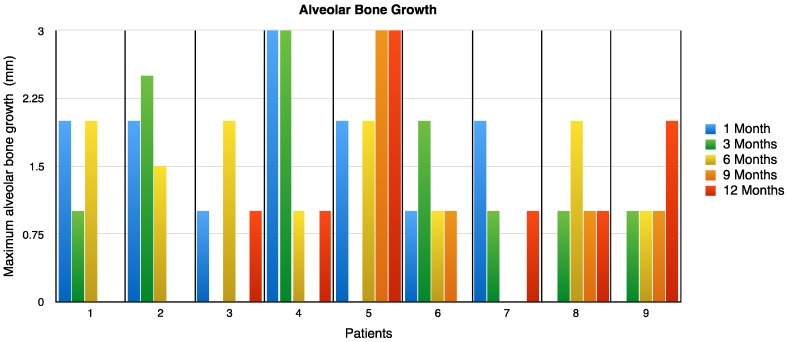
Maximum alveolar bone growth (mm) in periodontitis patients treated with the CH/HAP implant. Periodontitis patients treated with the CH/HAP implant were evaluated after the first month of treatment and subsequently every three months post-implant (pi), for a period of twelve months. Note that in patient 5, the highest level of alveolar bone growth (10 mm) occurred at twelve months pi (green bar) and some patients (1, 2, 3, 4 and 7) presented different alveolar bone growth after a period of six months pi. CH: Chitosan; HAP: Hydroxyapatite.

The alveolar bone growth was measured at 1, 3, 6, 9 and 12 months after treatment. This data is exhibited in (Figure 3). The values shown in the table for each patient observed must be added together to obtain the final growth; in patient number five, there was an increase of 10 mm at the end of the study.

**Figure 4:**
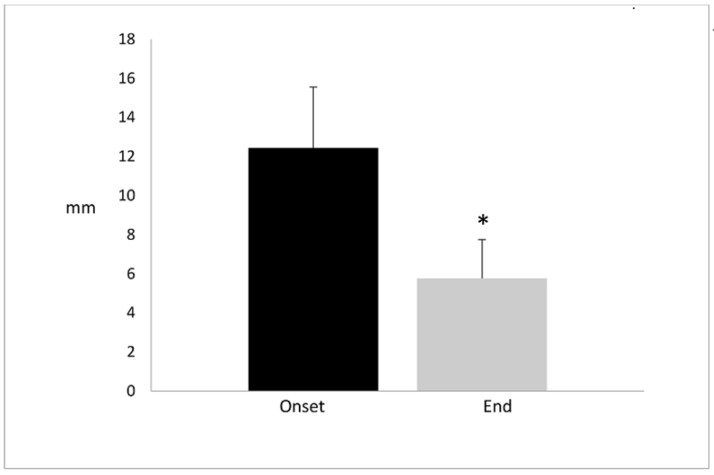
Alveolar bone size (mm) in local chronic periodontitis patients. The left bar represents the alveolar bone lost at the onset of the clinical study and the right bar represents the alveolar bone growth recovery at the end of the study. Data are expressed as the mean ± SD. The bone size defect at the onset of the study was larger than at the end of study with a statistically significant difference of * *p* < 0.001 (Student’s *t*-test).

In seven patients, the maximum alveolar bone growth was reached between the third and sixth month, post implant (pi). In two patients, alveolar bone grew beyond the sixth month, in one of the patients, it reached a maximal growth at the twelfth month. At the onset of the study, patients had a depth oral defect of 12.44 ± 3.12 mm without supporting bone. After a year of treatment, the depth defect was reduced to 5.77 ± 1.98. In other words, the bone grew about 6–7 mm. The oral defect reduction had a significant difference of *p* < 0.001 (Figure 4). 

**Figure 5:**
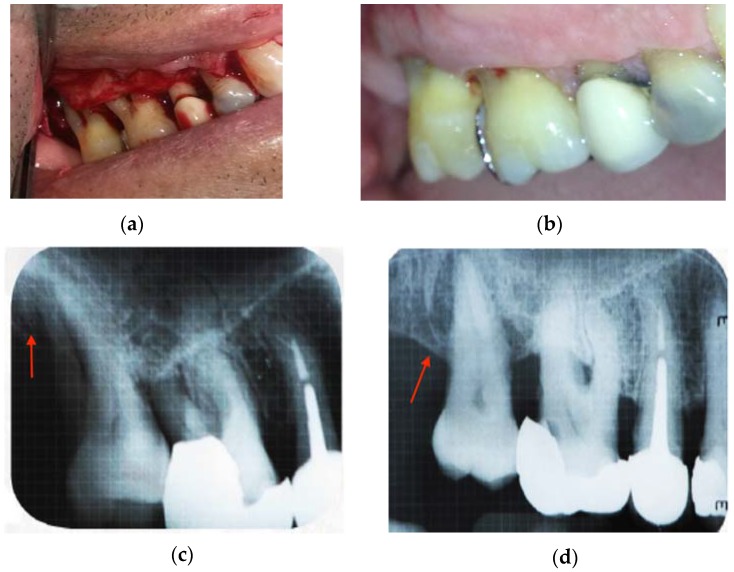
X-rays of one patient with local chronic periodontitis, at the onset of the CH/HAP implant and at the end of the treatment (12 months). The arrows show the alveolar bone growth (**c**) compared to the oral defect before the implant of the biomaterial (**d**). In (**a**) a considerable loss of mucous tissue is observed in the oral defect. In (**b**) a repair of mucous tissue on the implanted oral defect is observed. CH: Chitosan; HAP: Hydroxyapatite.

These results confirm that chitosan and hydroxyapatite treatment in chronic periodontitis patients promotes alveolar regeneration. Figure 5c,d shows the X-rays taken from one patient at the onset of the study and after one year of treatment with CH/HAP. Figure 5a exhibits the bone defect and Figure 5d shows the alveolar bone regeneration as indicated by an arrow. Figure 5b illustrates the recuperation of the gingival mucosa after treatment as compared with gingival mucosa before treatment Figure 5a. 

**Figure 6:**
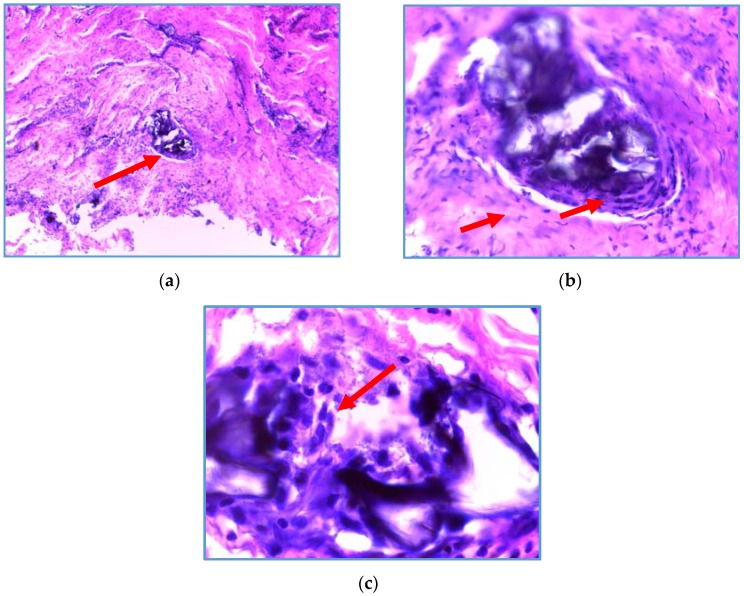
Histologic analysis of the biomaterial of CH/HAP taken three months after the implant in periodontitis patients. The biomaterial was stained with hematoxylin-eosin. In (**a**), the biomaterial is indicated by the arrow; the microphotography was taken with a 10× objective; in (**b**), the microphotography was taken at 40× amplification, where the collagen can be observed surrounding the biomaterial (pink color). The fibroblasts are stained in purple and are fusiform in shape, osteoblasts are purple rounded cells and they are infiltrated into biomaterial, these histological characteristics are indicated by the arrows; in (**c**), the biomaterial is stained in dark purple and the osteoblasts can be appreciated by the arrow. CH: Chitosan; HAP: Hydroxyapatite.

The biomaterial of CH/HAP was analyzed three months after the implant in periodontitis patients. The biopsy of the biomaterial exhibited a high activity of fibroblasts with a large content of collagen in the presence of osteoblasts and it did not have an inflammatory infiltrate (Figure 6).

**Figure 7:**
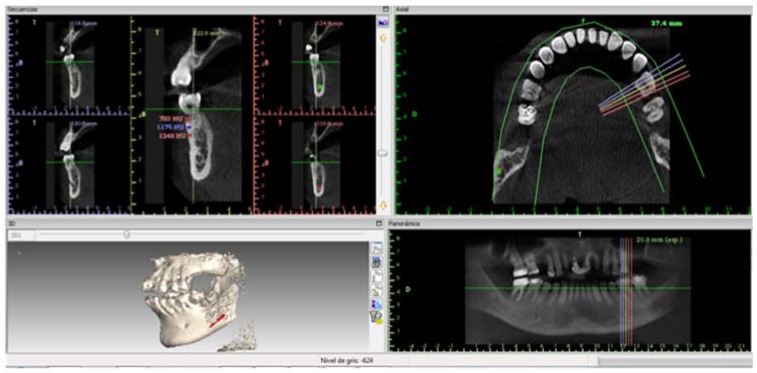
Image in 3D of one patient after the treatment was complimented. Bone density in tooth 37 in a treated patient is illustrated in the top left panel, five cuts were made every 2 mm, following the shape of the dental arch in order to evaluate the amount of bone by the vestibular side. Three measures were taken, one at the crest level, another in the middle zone and the third one at the apical level. The density is given in Hounsfield units. In the upper right panel a mandibular axial cut is illustrated. The 5 lines are shown as a reference to indicate the zone where the cuts were made. In the lower left panel, a 3D reconstruction of the oral cavity is shown. The lower right panel illustrates a panoramic lateral mandibular reconstruction of 20 mm wide.

Figure 7 illustrates bone density and includes the image of dental organ 37 from a periodontitis patient; the organ treated with CH/HAP exhibits an adequate density and growth compared to dental organ 36.

Table 1 shows the bone density in Hounsfield units in treated patients, Patient 1 had a higher density in the lateral superior right tooth treated than the central superior tooth, in the three measured zones: apical, middle and crest. In contrast, patient 2 treated with CH/HAP exhibited a lower density in the second right molar compared to the untreated first right molar. This result can be explained because the maxillary angle has a minor bone density. Patient 3 presented a higher bone density in the inferior first treated right molar than the untreated second right molar.

## 3. Discussion

In this pioneer clinical pilot study, we used an innovative procedure for the treatment of periodontitis by implanting CH/HAP. At the onset of the present study, the group of periodontitis patients had a significant oral defect with an important loss of alveolar bone of 12.44 mm; one-year post-implant the treated patients had an important alveolar bone growth of 5.77 ± 1.87 mm. This beneficial outcome for teeth preservation improved patient quality of life.

The biomaterial exhibited the presence of osteoblasts, fibroblasts and connective tissue without inflammatory infiltrate. The presence of osteoblasts seems to promote the alveolar bone growth filling the oral defect. 

The inorganic phase of the biomaterial integrated to the alveolar bone must be chemically and structurally similar to the mineral phase that forms the bone. In a previous study [10], the bioactivity of HAP and the presence of N-acetyl glucosamine in the chitosan analog of glycosaminoglycans (GAG), promoted bone growth [10]. When chitosan is mixed with hydroxyapatite it is difficult to achieve uniform distribution in the polymeric matrix. This situation sometimes limits its applicability, particularly with tissue inflammation, producing a voluminous fibrotic capsule and inducing a biomaterial rejection [11]. In this study, there was no such rejection, this could be attributed to an absence of an inflammatory infiltrate.

In diabetic rats with an oral defect, the oral inflammation was regulated using a combination of 2% Chitosan, type 1 collagen, and antibody anti-tumor necrosis factor α (anti-TNFα). In this implant, TNFα and the factor nuclear kappa B (NFkB) levels decreased. Furthermore, these implants produced an increase in osteogenesis which was associated with proteins that promoted the alveolar bone restoration [12]. In our results, Chitosan (3%) in combination with Hydroxyapatite (3%) promoted the alveolar bone growth. This combination of biomaterials could be inducing a decrease in the inflammation by the decrease in TNFα or NFkB. Additional studies should be made to corroborate this premise.

Huang et al. (2011) using an implant of chitosan/hydroxyapatite/collagen (CH/HAC) combined with allogeneic mesenchymal stem cells (MSC) in a rabbit’s femur bone defect, found larger areas of new bone and collagen fibrous tissues. The new bone was observed after twelve weeks post implant. The size and structure of alveolar bone in teeth is different to a large bone, therefore our scaffold is good enough to repair the lost alveolar bone [13].

Chitosan has an important microbicide activity [14,15,16,17]. Chitosan exhibits a potent oral plaque reduction action as well as an antibacterial activity against several oral pathogens such as Actinobacilus actinomycetemcomitans, Streptococcus mutans, and Porphyromonas gingivalis, all of which are implicated in plaque formation in periodontitis [18,19,20]. This antibacterial chitosan effect could also contribute to the repair of the oral defect. 

Hoemann (2005), found that chitosan hydrogels guide macrophages to the wounds, thus contributing to repair the wounds in vivo. Macrophages are also responsible for chitosan degradation in vivo through the release of cytokines as tumor necrosis factor α (TNFα) and interleukin-1β (IL-1 β), which attract fibroblast into the wound to initiate a reconstruction process. In the present study, chitosan was absorbed approximately in three months, with moderate alveolar bone growth. However, no macrophages were observed in the biomaterial analysis [21,22]. Chatelet (2001), showed that fibroblasts do not proliferate on chitosan films, independently of chitosan deacetylation grade, because of a great cellular adhesion to the biomaterial, thus inhibiting their proliferation [23]. In the present clinical study, the CH/HAP biomaterial was implanted in a paste. This biomaterial form may contribute to fibroblast proliferation and collagen formation and thus, induce the alveolar bone growth. 

The biomaterial of CH/HAP is cheaper than Emdogain, which supports its use in clinical odontology in low-income countries. Furthermore, the bone density was similar to that found in normal neighboring teeth, except for patient 2; this observation could be due to the position of the dental organ. In this case, the molar was in the maxillary angle, which has a spongier bone. 

The bone density in the oral defect treated with CH/HAP was larger in younger male patients than that in adult women patients. Perhaps this result is the consequence of calcium levels and is related to patient gender. This premise deserves to be confirmed.

## 4. Materials and Methods 

Nine patients with chronic periodontitis were studied, six men and three women, with ages ranging from 20 to 70 years old. All patients were treated in accordance with the 2013 Declaration of Helsinki. All patients agreed to participate in the study and signed the informed consent form.

### 4.1. Inclusion Criteria

Patients who exhibited localized chronic periodontitis with a pocket depth of 7–14 mm, accompanied by two wall intra-bone defects, without the presence of systemic diseases and who were non-smokers.

### 4.2. Exclusion Criteria

Patients with any periodontal treatment (one year before the study), pregnant women, and subjects with other chronic diseases, such as diabetes or cancer.

### 4.3. Elimination Criteria

Uncooperative study subjects and those individuals with any adverse reaction to the biomaterials.

### 4.4. Chitosan and Hydroxyapatite Implant

All patients presented localized chronic two-wall periodontitis with a vertical bone loss of seven millimeters or more. An implant of 3% chitosan and 3% hydroxyapatite was applied to each patient. Chitosan was obtained from Sigma-Aldrich with catalog number 448877. This molecule is 75–85% deacetylated, its viscosity is of 200–800 centipoise (cps), and it has a medium molecular weight. Hydroxyapatite (HAP) was obtained from Sigma with catalog number 289396. 3% deacetylated chitosan was dissolved in 10 ml of 2% acetic acid and then was mixed with 3% hydroxyapatite (HAP) under sterile conditions. That suspension was placed in a Petri cage. The material was dried for 24 h using sterile air into a laminar flow hood for 24 h. Later, 3 ml of saline physiologic solution (0.9%) was added to the CH/HAP to obtain a paste, the suspension had a final concentration of 0.0143 M. This paste was made immediately before making the implant for patients. The humidified CH/HAP paste was applied to the oral bone defect by filling the injured area completely. The membrane had the following size: 15.44 ± 4.79 mm large × 15.88 mm ± 2.50 high, with an average total surface of 245.18 mm^2^ and 1 mm^2^ of thickness; this size could change depending on the oral defect. The biomaterial elasticity, which was measured by a water column system, was of 82.3 ± 2.5 Kg and had a pressure of 58.3 ± 6.23 Pascals. Surgery was performed as follows: A full thickness flap was performed and elevated, followed by scaling and root planning in preparation; afterwards, the biomaterial was applied to the injured zone. The flap was closed and stitched up. Stitches were removed after 14 days.

Three months after the biomaterial implantation, a small sample was removed to study the distribution of fibroblasts, collagen, and osteoblasts and it was stained with hematoxylin-eosin. Ten sagittal cuts were obtained from the implanted biomaterial. The biomaterial was cut into 10 µm sections and these were mounted on slides. Hematoxylin solution was used for 5 min per slide and then alcoholic Eosin solution (0.5% eosin in 90% ethanol) was added for a period of 30 s. Then, slides were washed with deionized water for 5 min. Subsequently, an ethanol dehydration protocol was applied and the slides were covered with Entellan resin.

To measure the bone growth, a millimetric slide was used in the periapical radiography behind the tooth. Then, an X-ray was taken from each patient in the oral defect. The X-ray image was measured from the crown to the root of the tooth. To evaluate the level of teeth insertion, mucosa clinical photographs were taken of each patient. Periodontal probes with William´s markings at 1, 2, 3, 5, 7, 8, 9 and 10 mm were used to measure the pocket deep by introducing this instrument between the mucosa and the tooth.

To evaluate the tooth mobility grade, we employed a dental mirror and a dental tweezer generating a weak pressure in a horizontal direction. Then, we used the classification of dental mobility from 0 to 3 of American Academy of Periodontology. 

To analyze the bone density of the oral defect from periodontitis patients treated with CH/HAP, a Computerized Tomography was taken and analyzed with the software Implant Viewer from Anne Solutions Company using the lateral tooth density as a control. 

### 4.5. Statistics

All data are expressed as Mean ± SE. The U-Mann Whitney test was used to compare data among patients. To compare the data of alveolar bone growth among patients a paired Student’s *t*-test was used.

## 5. Conclusions

The CH/HAP implant is a therapeutic strategy for chronic periodontitis patients that allows guided bone regeneration at a low cost, expanding the opportunity of their use for people in countries with a low socioeconomic grade.

The CH/HAP implant reduced the pocket depth of the supporting tissue. It also reduced the grading of tooth mobility and promoted alveolar bone growth.

The patients conserved the dental organ, favoring a better quality of life.

It is important to highlight that there was no inflammation surrounding the implant.

## Figures and Tables

**Table 1:** The bone density in Hounsfield (UH) units in patients treated with a CH/HAP implant. The bone density in UH units of dental organs (do) 12, 17 and 36 compared to do 11, 16 and 37 respectively, is shown. The bone density was measured in three zones: apical, middle and crest. Note that patients 1 and 3 had a higher density in the oral bone defect compared with the untreated nearest tooth. Patient 2 had a low density in the treated oral defect compared to the nearest untreated tooth. All data were obtained at one year of treatment. CH: Chitosan; HAP: Hydroxyapatite.

Patients	Tooth	Hounsfield (UH) Apical	UH Middle	Crest
Patient 1	Tooth #12 (Treated)	1201	1080	587
	Tooth #11	1044	647	451
Patient 2	Tooth #16	914	814	409
	Tooth #17 (Treated)	729	632	285
Patient 3	Tooth #36 (Treated)	893	1334	944
	Tooth #37	801	944	747

## References

[1] Majzoub Z., Bobbo M., Atiyeh F., Cordioli G. (2005). Two patterns of histologic healing in an intrabony defect following treatment with enamel matrix derivative: A human case report. Int. J. Periodontics Restor. Dent..

[2] Sculean A., Windisch P., Keglevich T., Chiantella G.C., Gera I., Donos N. (2003). Clinical and Histologic Evaluation of Human Intrabony Defects Treated with an Enamel Matrix Protein Derivative Combined with a Bovine-Derived Xenograft. Int. J. Periodontics Restor. Dent..

[3] Bernabéu Martínez E., López-Oliva Muñoz F., Larena Pellejero A., Tur Gil A., de la Piedra Gordo M.C., Montero Escobar M. (2006). Estudio de la composición ósea para su apropiada regeneración con materiales implantados. Patología del Aparato Locomotor.

[4] Jun S.H., Lee E.J., Jang T.S., Kim H.E., Jang J.H., Koh Y.H. (2013). Bone morphogenic protein-2 (BMP-2) loaded hybrid coating on porous hydroxyapatite scaffolds for bone tissue engineering. J. Mater. Sci. Mater. Med..

[5] Ueno H., Nakamura F., Murakami M., Okumura M., Kadosawa T., Fujinaga T. (2001). Evaluation effects of chitosan for the extracellular matrix production by fibroblasts and the growth factors production by macrophages. Biomaterials.

[6] Yamaguchi I., Iizuka S., Osaka A., Monma H., Tanaka J. (2003). The effect of citric acid addition on chitosan/hydroxyapatite composites. Coll. Surf. A.

[7] Ezoddini-Ardakani F., Navabazam A., Fatehi F., Danesh-Ardekani M., Khadem S., Rouhi G. (2012). Histologic evaluation of chitosan as an accelerator of bone regeneration in microdrilled rat tibias. Dent. Res. J..

[8] Meseguer-Olmo L., Muñoz-Ruiz J., Bernabeu-Esclapez A., Clavel-Sainz Nolla M., Arcos-Pérez D., Vallet-Regí M., López-Prats F., Lax-Pérez A., Meseguer-Ortiz de Villajos C.L. (2006). Cinética de crecimiento in vitro de osteoblastos humanos sobre cerámica porosa de hidroxiapatita. ROT Revista de Ortopedia y Traumatología.

[9] de la Concepción Matesanz M., Feito M.J., Ramírez-Santillán C., Lozano R.M., Sánchez-Salcedo S., Arcos D., Vallet-Regí M., Portolés M.T. (2012). Signaling pathways of immobilized FGF-2 on silicon-substituted hydroxyapatite. Macromol. Biosci..

[10] Wu J.Q., Liu Y., Yang T.F., Mu Y.H., Guo T., Li Y.B. (2007). Porous polyvinyl alcohol hydrogel composite prepared and studied initially for biocompatibility. J. Sichuan Univ. Med. Sci. Ed..

[11] Davidenko N., García R., Peniche C., Solís Y. (2010). Chitosan/hydroxyapatite-based composites. Biotecnología Aplicad.

[12] Wang Q., Li H., Xiao Y., Li S., Li B., Zhao X., Ye L., Guo B., Chen X., Ding Y., Bao C. (2015). Locally controlled delivery of TNFa antibody from a novel glucose-sensitive scaffold enhances alveolar bone healing in diabetic conditions. COREL J. Control. Release.

[13] Huang Z., Chen Y., Feng Q.-L., Zhao W., Yu B., Tian J., Li S.-J., Lin B.-M. (2011). In vivo bone regeneration with injectable chitosan/hydroxyapatite/collagen composites and mesenchymal stem cells. Front. Mater. Sci..

[14] Martinez L.R., Mihu M.R., Han G., Frases S., Cordero R.J., Casadevall A., Friedman A.J., Friedman J.M., Nosanchuk J.D. (2010). The use of chitosan to damage Cryptococcus neoformans biofilms. Biomaterials.

[15] Muzzarelli R., Tarsi R., Filippini O., Giovanetti E., Biagini G., Varaldo P.E. (1990). Antimicrobial properties of N-carboxybutyl chitosan. Antimicrob. Agents Chemother..

[16] Rhoades J., Roller S. (2000). FOOD MICROBIOLOGY—Antimicrobial Actions of Degraded and Native Chitosan against Spoilage Organisms in Laboratory Media and Foods. Appl. Environ. Microbiol..

[17] Jeon Y.-J., Park P.-J., Kim S.-K. (2001). Antimicrobial effect of chitooligosaccharides produced by bioreactor. Carbohydr. Polym..

[18] No H.K., Park N.Y., Lee S.H., Meyers S.P. (2002). Antibacterial activity of chitosans and chitosan oligomers with different molecular weights. Int. J. Food Microbiol..

[19] Choi B.K., Kim K.Y., Yoo Y.J., Oh S.J., Choi J.H., Kim C.Y. (2001). In vitro antimicrobial activity of a chitooligosaccharide mixture against Actinobacillus actinomycetemcomitans and Streptococcus mutans. Int. J. Antimicrob. Agents.

[20] Ikinci G., Senel S., Akincibay H., Kas S., Ercis S., Wilson C.G., Hincal A.A. (2002). Effect of chitosan on a periodontal pathogen Porphyromonas gingivalis. Int. J. Pharm..

[21] Hoemann C.D., Sun J., Légaré A., McKee M.D., Buschmann M.D. (2005). Tissue engineering of cartilage using an injectable and adhesive chitosan-based cell-delivery vehicle. Osteoarthr. Cartil..

[22] Chellat F., Tabrizian M., Dumitriu S., Chornet E., Magny P., Rivard C.H., Yahia L. (2000). In vitro and in vivo biocompatibility of chitosan-xanthan polyionic complex. J. Biomed. Mater. Res..

[23] Chatelet C., Damour O., Domard A. (2001). Influence of the degree of acetylation on some biological properties of chitosan films. Biomaterials.

